# SDF‐1 Attenuates Oocyte Quality Decline During Reproductive Aging Through Autophagy‐Enhanced Stress Granule Scavenging

**DOI:** 10.1002/advs.76902

**Published:** 2026-08-03

**Authors:** Rui Long, Meng Wang, Ruolin Mao, Hong Chen, Juepu Zhou, Xiangfei Wang, Zheng Yuan, Ri‐Cheng Chian, Nan Xiao, Qingsong Xi, Yimin Shu, Lei Jin, Lixia Zhu

**Affiliations:** ^1^ Reproductive Medicine Center, Tongji Hospital, Tongji Medical College Huazhong University of Science and Technology Wuhan China; ^2^ Institute of Chinese Materia Medica China Academy of Chinese Medical Sciences Beijing China; ^3^ Center For Reproductive Medicine Heilongjiang Province Hospital Harbin China; ^4^ Department of Center for Reproductive Medicine Tianjin Central Hospital of Obstetrics and Gynecology/Nankai University Affiliated Maternity Hospital/Tianjin Key Laboratory of Human Development and Reproductive Regulation Tianjin China; ^5^ Center of Oncology, Tongji Hospital, Tongji Medical College Huazhong University of Science and Technology Wuhan China; ^6^ Advanced Reproductive Medicine, Department of Obstetrics and Gynecology University of Kansas Medical Center Kansas City Kansas USA

**Keywords:** aging, autophagy, oocyte quality, SDF‐1, stress granule

## Abstract

The age‐related decline in oocyte quality constitutes a major cause of reduced female fertility. In this study, we investigated the potential of stromal cell‐derived factor‐1 (SDF‐1) to counteract oocyte deterioration during reproductive aging. We observed a significant negative correlation between SDF‐1 levels and aging in both human follicular fluid/oocytes and murine ovarian tissues. Utilizing aged mouse models, we found that SDF‐1 supplementation, both in vitro and in vivo, was associated with the amelioration of multiple aging‐associated oocyte defects, including restored meiotic spindle morphology, improved chromosomal alignment, normalized distribution of cortical granules and mitochondria, enhanced mitochondrial membrane potential, and reduced oxidative stress. Consequently, SDF‐1 treatment improved fertilization competence, embryonic developmental potential, and fertility restoration in aged female mice. Mechanistically, transcriptomic and functional analyses suggested that SDF‐1 ameliorates oocyte aging primarily by enhancing autophagic activity, which was associated with clearance of accumulated stress granules and mitigation of oxidative damage. Pharmacological inhibition of autophagy attenuated the beneficial effects of SDF‐1. In conclusion, our findings point to a previously underexplored role for SDF‐1 in alleviating age‐related oocyte decline, potentially through autophagy‐enhanced stress granule scavenging, positioning SDF‐1 as a promising candidate for therapeutic intervention in reproductive aging, although further investigations are warranted in the future.

## Graphical abstract

SDF‐1 levels decline significantly with maternal aging. Exogenous supplementation restores meiotic spindle morphology, chromosomal alignment, and mitochondrial function while reducing oxidative stress in aged oocytes. Mechanistically, SDF‐1 enhances autophagic activity to clear accumulated stress granules, thereby rescuing fertilization competence and restoring fertility in aged mice, offering a novel therapeutic strategy for reproductive aging.

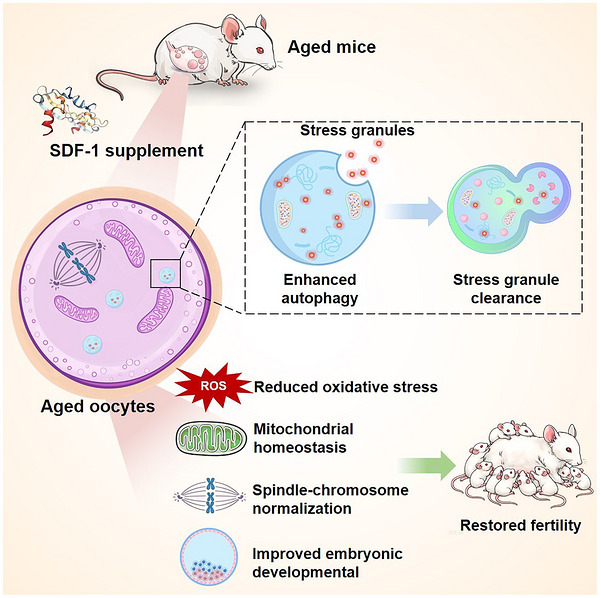

## 1 Introduction

The age‐related decline in female fertility represents a significant clinical challenge in reproductive medicine. This decline becomes particularly evident beyond 35 years old, as the rate of natural infertility rises dramatically from approximately 10% in women under 34 to nearly 90% after age 45 [1]. Although assisted reproductive technology (ART) offers a way to conception, its efficacy is deeply compromised by advanced maternal age [2]. It has been reported that live birth rates following in vitro fertilization (IVF) exceed 40% for women under 35 but sharply decrease to merely 1%–2% by age 44 [3]. This reproductive senescence is largely attributable to a reduction in ovarian reserve and a deterioration in oocyte quality, often accompanied by increased aneuploidy [4].

The deterioration of oocyte quality during reproductive aging is characterized by a series of profound cellular and molecular alterations. Morphologically and functionally, aged oocytes frequently exhibit severe mitochondrial dysfunction, which is characterized by decreased ATP production and the pathological accumulation of reactive oxygen species (ROS). This severe oxidative stress disrupts intracellular redox homeostasis and directly impairs the meiotic apparatus, leading to aberrant spindle assembly, chromosome misalignment, and a significantly elevated incidence of aneuploidy [5]. Consequently, these intrinsic defects severely compromise the fertilization capacity and subsequent embryonic developmental potential of aged oocytes.

To counteract the age‐related decline in ovarian function and oocyte quality, several clinical and preclinical interventions have been actively explored. Antioxidant therapies, such as coenzyme Q10, resveratrol, nicotinamide mononucleotide (NMN), melatonin, and vitamin E, have been widely applied, effectively alleviating oxidative damage and improving aged oocyte quality [5, 6]. Moreover, the administration of specific hormones and growth factors, including vascular endothelial growth factor (VEGF), insulin‐like growth factor 1 (IGF‐1), and growth hormone (GH), has shown benefits in modulating the ovarian microenvironment and promoting follicular developmentc [7]. Furthermore, intraovarian injection of platelet‐rich plasma (PRP) or plasma rich in growth factors (PRGF) has recently gained attention as an innovative regenerative approach in improving ovarian function and oocyte quality, although large‐scale randomized controlled trials are still required to definitively validate its clinical efficacy [8]. Although the above‐mentioned interventions have shown promise in mitigating these defects, identifying more potential therapeutic strategies remains an urgent research priority.

Stromal cell‐derived factor‐1 (SDF‐1), also known as C‐X‐C motif chemokine 12 (CXCL12), is a multifunctional cytokine known to regulate diverse cellular processes, including antioxidant defense, migration, proliferation, and differentiation, across various cell types [9]. C‐X‐C Chemokine Receptor 4 (CXCR4) and C‐X‐C Chemokine Receptor 7 (CXCR7) have been well characterized as the binding partners of SDF‐1 [10]. The SDF‐1/CXCR4 axis, in particular, is critical for implantation and placentation [11]. Accumulating evidence suggests important roles for SDF‐1 in female fertility and early embryo development. For instance, the SDF‐1/CXCR4 pathway has been shown to recruit bone marrow‐derived mesenchymal stem cells to facilitate endometrial repair [12]. Regarding oocyte maturation, clinical observations indicate that SDF‐1 concentrations in human follicular fluid positively correlate with follicular diameter and follicular fluid volume, suggesting its potential as a biochemical marker of oocyte quality [13]. Experimental studies further support that supplementation of SDF‐1 during in vitro maturation of porcine oocytes enhanced their developmental competence by modulating the transcription of genes related to purine metabolism and oocyte maturation [14]. Similarly, in sheep and porcine models, the inclusion of SDF‐1 in maturation media improved subsequent embryonic development [14, 15]. The physiological indispensability of SDF‐1/CXCR4 signaling is further highlighted by the embryonic lethality observed in SDF‐1 or CXCR4 deficiency mice [16]. Moreover, Jaleel et al. revealed that SDF‐1/CXCR4 signaling promotes trophoblast survival via anti‐apoptotic mechanisms during pregnancy [17]. Despite these established roles in reproductive processes, the potential of SDF‐1 to mitigate the decline in oocyte quality associated with maternal aging remains incompletely understood.

In this study, we systematically evaluated the potential of SDF‐1 to counteract female reproductive aging using a multi‐level approach that included clinical specimen analyses and established mouse models of reproductive aging. Our results showed that SDF‐1 supplementation, both in vitro and in vivo, improved oocyte quality and fertility outcomes in aged female mice. To explore the underlying mechanisms, we performed transcriptomic profiling and a series of molecular and cellular experiments, which suggested that the beneficial effects of SDF‐1 may involve enhanced autophagy‑mediated clearance of stress granules in aged oocytes. These findings offer new insights into how SDF‑1 may ameliorate age‑associated oocyte deterioration and support its further investigation as a potential candidate for therapeutic intervention in reproductive aging.

## 2 Results

### 2.1 SDF‐1 Levels are Negatively Correlated With Ovarian Aging

**FIGURE 1:**
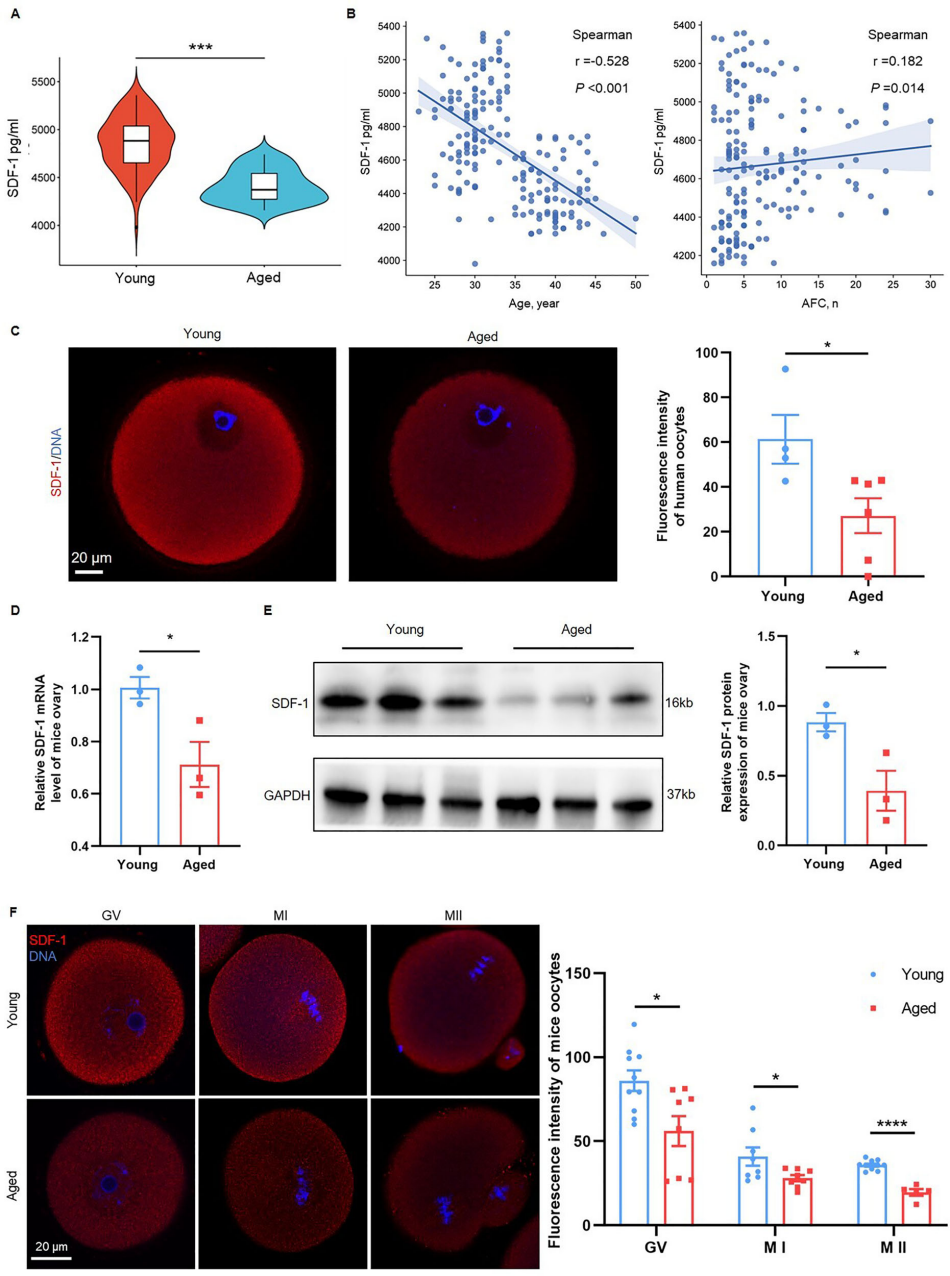
The level of SDF‐1 negatively correlates with age‐related ovarian aging. (A) The concentration of SDF‐1 in human follicular fluid (FF). (B) Spearman correlation of association between the concentration of SDF‐1 in human FF and female age, as well as antral follicle count (AFC). (C) Representative images and relative fluorescence intensity of SDF‐1 in oocytes from young and aged females. (D) The mRNA level of SDF‐1 in the ovaries from young and aged mice. (E) The protein expression of SDF‐1 in the ovaries from young and aged mice. (F) Representative images and relative fluorescence intensity of SDF‐1 in various stages of oocytes from young and aged mice. Red, SDF‐1; blue, DNA. FF, follicular fluid; AFC, antral follicle count; GV, germinal vesicle; MI, metaphase I; MII, metaphase II. *, *p <* 0.05; ***, *p <* 0.001; ****, *p <* 0.0001.

Follicular fluid (FF) was collected from women undergoing IVF, and concentrations of SDF‐1 were measured. It was shown that SDF‐1 levels in FF from younger patients (<35 years) were significantly higher than those from women of advanced (≥35 years) (4853±27.3 vs. 4410±19.7 pg/mL, *p* < 0.0001, Figure 1A) In addition, a significant correlation was observed between SDF‐1 concentration in human FF and female age (*p* < 0.001), as well as with antral follicle count (AFC, *p* = 0.014), a key indicator of ovarian reserve (Figure 1B). Immunofluorescence staining also revealed higher SDF‐1 expression in germinal vesicle (GV) oocytes from younger women (<28 years) compared to those from older patients (>35 years) (Figure 1C). Consistent with these human data, SDF‐1 expression in murine reproductive tissues showed a similar pattern. Both mRNA (Figure 1D) and protein (Figure 1E) levels of SDF‐1 were higher in ovaries from young mice compared to older ones. Notably, regardless of meiotic stage, oocytes from younger mice exhibited significantly stronger SDF‐1 fluorescence intensity than those from the older (*p* = 0.012 for GV; *p* = 0.043 for MI; *p* < 0.001 for MII oocytes; Figure 1F).

### 2.2 SDF‐1 Improves the Oocyte Quality of Aged Mice In vitro

**FIGURE 2:**
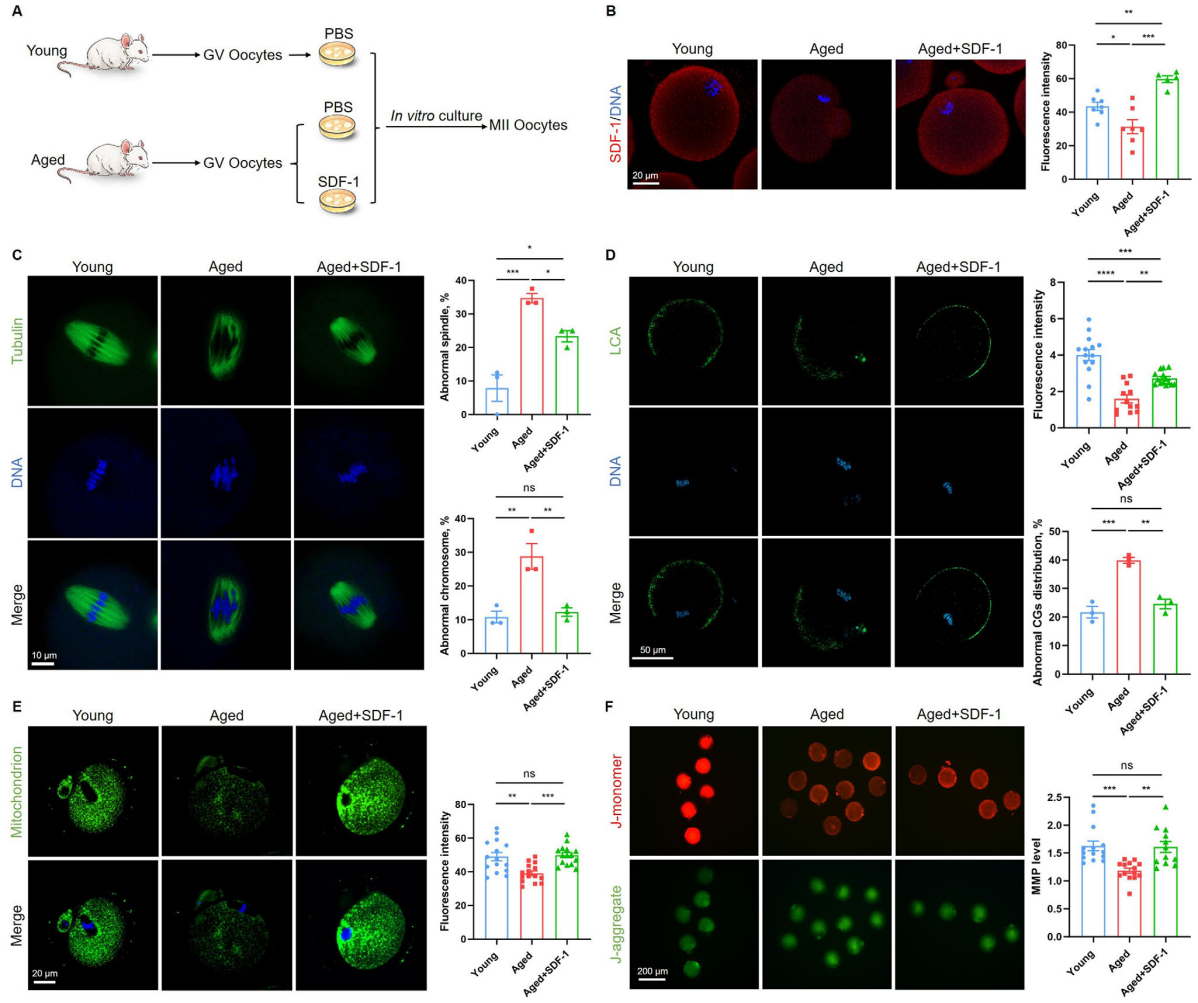
SDF‐1 supplementation enhances the quality of aged mice oocytes in vitro. (A) The schematic diagram of in vitro treatment of aged mice oocytes with SDF‐1. (B) Representative images and relative fluorescence intensity of SDF‐1 in mice oocytes from the young, aged, and aged+SDF‐1 group. (C) Representative images and the percentage of aberrant spindles and misaligned chromosomes in mice oocytes from the young, aged, and aged+SDF‐1 group. (D) Representative images, relative fluorescence intensity and the percentage of abnormal cortical granules (CGs) distribution in mice oocytes from young, aged and aged+SDF‐1 group. (E) Representative images and fluorescence intensity of mitochondrion in mice oocytes from young, aged and aged+SDF‐1 group. (F) Representative images and fluorescence intensity of mitochondrial membrane potential as assessed by JC‐1 staining in mice oocytes from young, aged and aged+SDF‐1 group. PMSG, pregnant mare serum gonadotropin; GV, germinal vesicle; MII, metaphase II; CG, cortical granule. *, *p <* 0.05; **, *p <* 0.01; ***, *p <* 0.001; ****, *p <* 0.0001; ns, not significant.

GV oocytes were collected from both young and aged mice and cultured in vitro to the MII stage in the presence or absence of SDF‐1. A schematic of the SDF‐1 supplementation regimen is provided (Figure 2A). Oocytes were cultured in medium containing different concentrations of SDF‐1, and the maturation rate was recorded and compared across groups, with an optimal concentration of 20 ng/mL identified in subsequent experiments. (Table S3). Following treatment, oocytes from aged mice exhibited elevated SDF‐1 levels, suggesting increased local accumulation in vitro (Figure 2B). Although the rates of GVBD were comparable across groups, the PB1 extrusion rate was significantly higher in oocytes from aged mice after SDF‐1 treatment (Figure S1B). To further evaluate the effect of SDF‐1 on oocytes, the quality of MII oocytes was examined. Oocytes from young mice typically displayed a barrel‐shaped meiotic spindle with well‐focused poles and tightly aligned chromosomes at the metaphase plate. In contrast, oocytes from aged mice frequently showed disorganized spindles with irregular contours. SDF‐1 treatment significantly reduced the incidence of both spindle abnormalities and chromosomal misalignment in aged oocytes (Figure 2C), suggesting a restorative effect of SDF‐1 on age‐related meiotic defects. Beyond nuclear maturation, cytoplasmic quality, assessed via CGs distribution and mitochondrial organization, was also evaluated. In normal oocytes, CGs form a continuous subcortical layer excluding the nuclear area. Aged oocytes showed markedly diminished CG signals, which were largely restored by SDF‐1 supplementation (Figure 2D). Similarly, SDF‐1 ameliorated mitochondrial abnormalities in aged oocytes. In high‐quality oocytes from young mice, mitochondria were evenly distributed and accumulated in the perinuclear region. This distribution pattern was disrupted in aged oocytes but recovered following SDF‐1 treatment (Figure 2E). Moreover, the age‐related decline in mitochondrial membrane potential was rescued by SDF‐1 supplementation (Figure 2F).

### 2.3 SDF‐1 Recovers Fertility and Oocyte Quality of Aged Mice In Vivo

**FIGURE 3:**
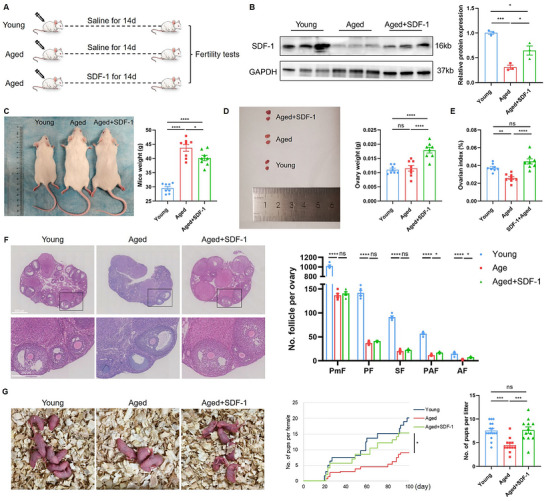
The in vivo supplementation of SDF‐1 improves the fertility of aged mice. (A) The schematic diagram of in vivo treatment of aged mice with SDF‐1/saline. Fertility tests were performed after 14‐day treatment. (B) The protein expression of SDF‐1 in the mice ovaries from the young, aged, and aged+SDF‐1 group. (C) Representative images and the weight of the mice in each group. (D) Representative images and the ovary weight in each group. (E) The ovarian index in the mice from the young, aged, and aged+SDF‐1 group. (F) Representative images of mice ovarian morphology and the ovarian follicles count at different developmental stages in young, aged and aged+SDF‐1 ovaries. (G) Representative images of pups delivered by young, aged, and aged+SDF‐1 group, and the number of pups per female as well as litter size over time for 14 weeks after mating with young male mice in each group. PmF, primordial follicle; PF, primary follicle; SF, secondary follicle; PAF, pre‐antral follicle; AF, antral follicle. *, *p <* 0.05; **, *p <* 0.01; ***, *p <* 0.001; ****, *p <* 0.0001; ns, not significant.

To evaluate the impact of SDF‐1 on fertility of aged mice in vivo, young and aged mice received daily intraperitoneal administration of either saline or SDF‐1 (10 µg/kg) for 14 days (Figure 3A). Following the treatment, ovarian SDF‐1 protein levels were significantly elevated in aged mice, demonstrating a robust increase in local target engagement (Figure 3B). As expected, body weight increased with age in mice. Although absolute ovary weight remained comparable between young and aged mice, the ovarian index (ovary weight to body weight ratio) was significantly reduced in aged animals. Interestingly, SDF‐1 treatment mitigated the age‐associated increase in body weight (Figure 3C) and increased ovary weight in aged mice (Figure 3D), resulting in a significantly higher ovarian index compared to untreated aged controls (Figure 3E). In addition, we quantified follicle numbers at different developmental stages. It was found that SDF‐1 administration increased the total number of follicles per ovary, with a particularly pronounced effect on pre‐antral and antral follicles (Figure 3F), indicating that SDF‐1 can rescue ovarian reserve decrease in aged mice. Furthermore, aged mice receiving SDF‐1 delivered more pups per female (15.3 vs. 9.0, *p* = 0.015, Figure 3G) and exhibited a higher number of pups per litter (7.7±0.7 vs. 4.5±0.4, *p <* 0.001, Figure 3G), demonstrating enhanced fertility following SDF‐1 intervention.

**FIGURE 4:**
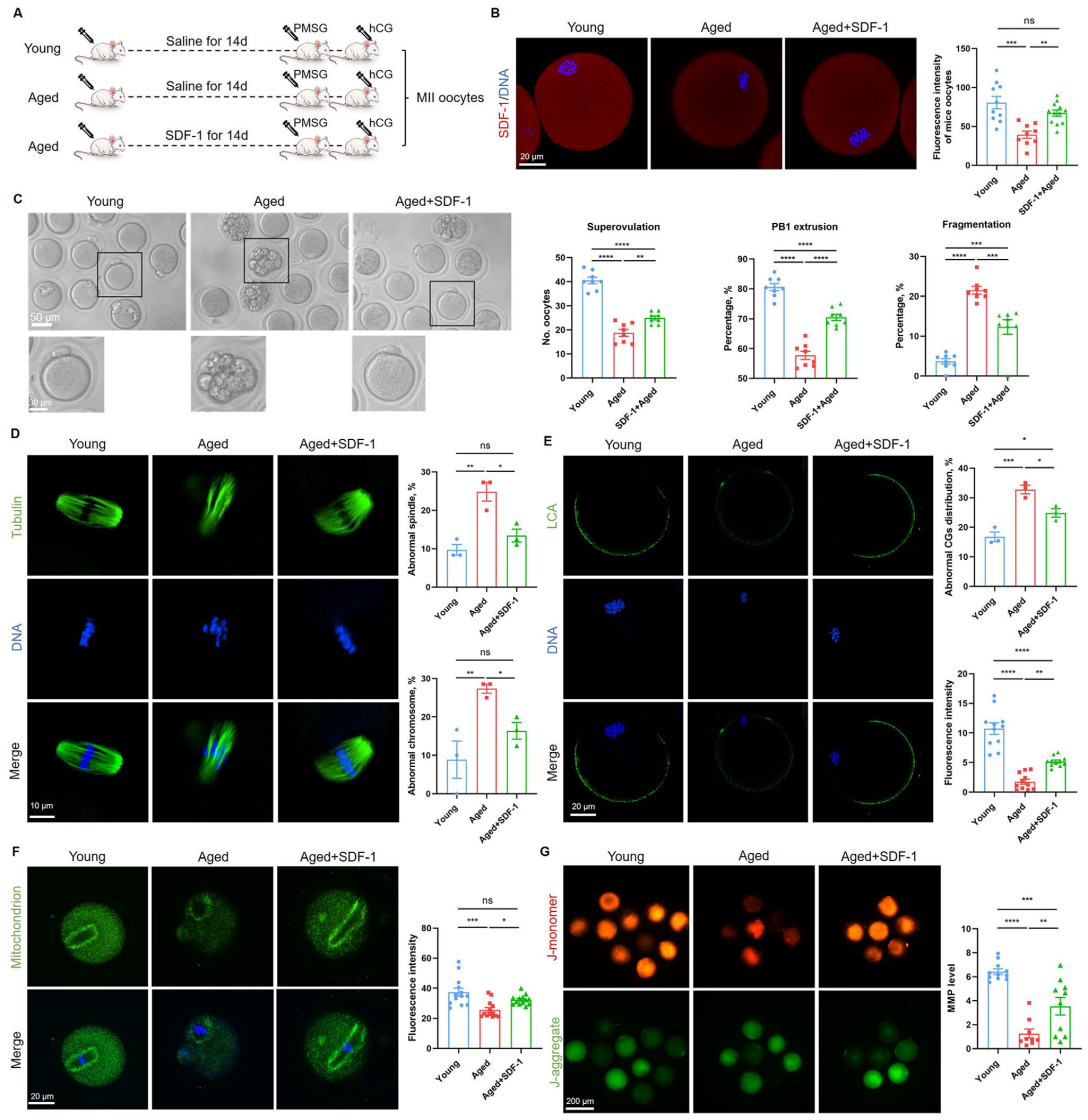
The in vivo supplementation of SDF‐1 enhances the oocytes quality of aged mice. (A) The schematic diagram of in vivo treatment of aged mice with SDF‐1/saline. The quality of MII oocytes were assessment after 14‐day treatment. (B) Representative images and relative fluorescence intensity of SDF‐1 in the mice MII oocytes from different groups. (C) Representative images of oocytes, the number of ovulated oocytes, the percentage of PB1 extrusion and the percentage of oocyte fragmentation in young, aged and aged+SDF‐1 mice. (D) Representative images, the percentage of aberrant spindles and the misaligned chromosomes in mice MII oocytes from the young, aged and aged+SDF‐1 group. (E) Representative images, relative fluorescence intensity, and the percentage of abnormal cortical granules (CGs) distribution in mice oocytes from the young, aged and aged+SDF‐1 group. (F) Representative images and fluorescence intensity of mitochondrion in mice MII oocytes from young, aged and aged+SDF‐1 group. (G) Representative images and fluorescence intensity of mitochondrial membrane potential as assessed by JC‐1 staining in mice MII oocytes from young, aged and aged+SDF‐1 group. PMSG, pregnant mare serum gonadotropin; hCG, human chorionic gonadotropin; MII, metaphase II; PB, polar body; CG, cortical granule. *, *p <* 0.05; **, *p <* 0.01; ***, *p <* 0.001; ****, *p <* 0.0001; ns, not significant.

Subsequently, MII oocytes were collected from different groups after superovulation (Figure 4A). SDF‐1 administration resulted in elevated SDF‐1 protein levels in oocytes from aged mice (Figure 4B), thereby confirming the effectiveness of intracellular SDF‐1 enrichment. As anticipated, aging was found to be associated with a reduced oocyte number following superovulation, a decreased PB1 extrusion rate, and an increased incidence of oocyte fragmentation. These age‐related defects could be rescued by SDF‐1 supplementation to a certain extent (Figure 4C). Consistent with the in vitro supplementation results, in vivo SDF‐1 injection significantly improved nuclear maturation in aged oocytes. This was evidenced by a significant reduction in the proportion of oocytes with aberrant spindle morphology and chromosome misalignment (Figure 4D). Moreover, SDF‐1 treatment restored the normal subcortical distribution pattern of CGs, which was impaired in oocytes from aged mice (Figure 4E). Regarding mitochondrial function, SDF‐1 promoted the restoration of the perinuclear accumulation of mitochondria (Figure 4F) and increased the mitochondrial membrane potential in aged oocytes (Figure 4G). Furthermore, in vivo administration of SDF‐1 enhanced sperm binding affinity to the ZP of oocytes from aged mice, indicating improved fertilization competence (Figure S2A). SDF‐1 treatment also significantly enhanced the developmental potential of embryos derived from aged oocytes, as demonstrated by increased rates of fertilization, cleavage, and blastocyst formation (Figure S2B).

### 2.4 SDF‐1 Ameliorates Oxidative Stress in Oocytes From Aged Mice

**FIGURE 5:**
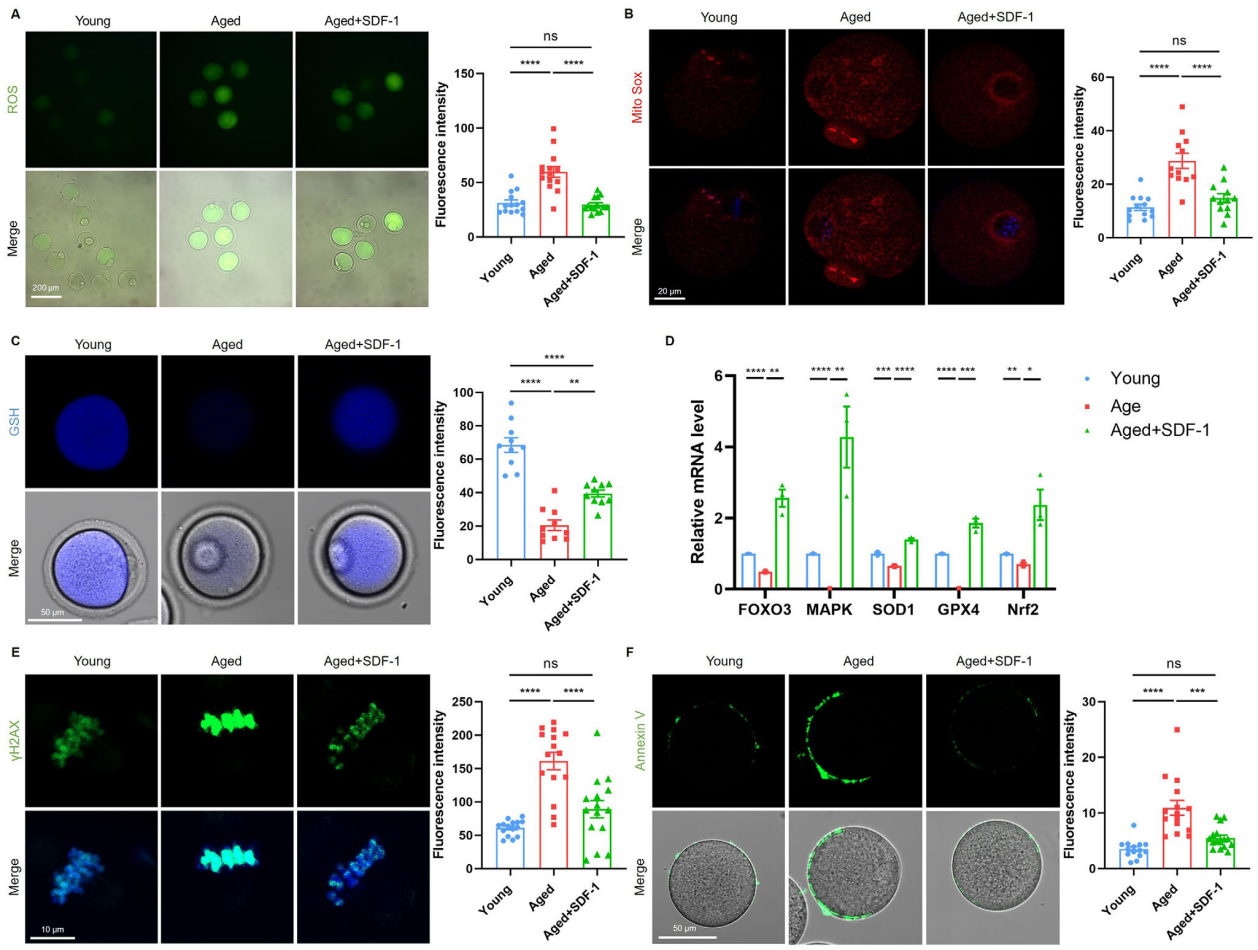
The supplementation of SDF‐1 decreases the oxidative stress level and maintains redox balance in aged mice oocytes. (A) Representative images and fluorescence intensity of ROS levels in oocytes from young, aged, and aged+SDF‐1 group. (B) Representative images and fluorescence intensity of superoxide levels as detected by Mito Sox staining in oocytes from young, aged, and aged+SDF‐1 group. (C) Representative images and fluorescence intensity of GSH level in oocytes from young, aged, and aged+SDF‐1 mice. (D) The mRNA level of antioxidant‐related genes in oocytes from young, aged, and aged+SDF‐1 mice. (E) Representative images of γH2AX fluorescence signals and relative fluorescence intensity of γH2AX in oocytes from young, aged and aged+SDF‐1 mice. (F) Representative images and relative fluorescence intensity of Annexin V in oocytes from young, aged, and aged+SDF‐1 mice. *, *p <* 0.05; **, *p <* 0.01; ***, *p <* 0.001; ****, *p <* 0.0001; ns, not significant.

Oxidative stress is a well‐known factor that impairs oocyte quality. ROS levels were measured in MII oocytes following in vitro culture. Supplementing the culture medium with SDF‐1 significantly reduced ROS levels in oocytes from aged mice (Figure 5A), indicating a mitigation of oxidative imbalance. Consistently, Mito SOX staining revealed a marked decrease in superoxide levels upon SDF‐1 treatment (Figure 5B). A similar reduction in ROS and Mito SOX was observed in oocytes following in vivo SDF‐1 administration (Figure S3A,B). Furthermore, intracellular GSH levels were significantly elevated in aged oocytes after SDF‐1 supplementation (Figure 5C), reinforcing the antioxidant role of SDF‐1. Analysis of antioxidant gene expression showed that SDF‐1 treatment significantly upregulated the mRNA levels of key antioxidant genes in oocytes from aged animals (Figure 5D). Given that excessive ROS can induce DNA damage, we evaluated DNA double‐strand breaks by immunofluorescence staining of γH2AX. As expected, aged oocytes exhibited higher γH2AX intensity compared to young oocytes, and this increase was attenuated by SDF‐1 supplementation (Figure 5E). In addition, Annexin‐V staining demonstrated a significant increase in apoptosis in aged oocytes, which was also rescued by SDF‐1 treatment (Figure 5F). Taken together, advanced maternal age leads to excessive ROS accumulation, resulting in elevated DNA damage and apoptosis. SDF‐1 supplementation counteracts these effects by enhancing the antioxidant capacity, thereby effectively alleviating maternal aging‐induced oxidative stress.

### 2.5 SDF‐1 Remodels the Oocyte Transcriptome to Counteract Aging‐Associated Decline in Oocyte Quality

**FIGURE 6:**
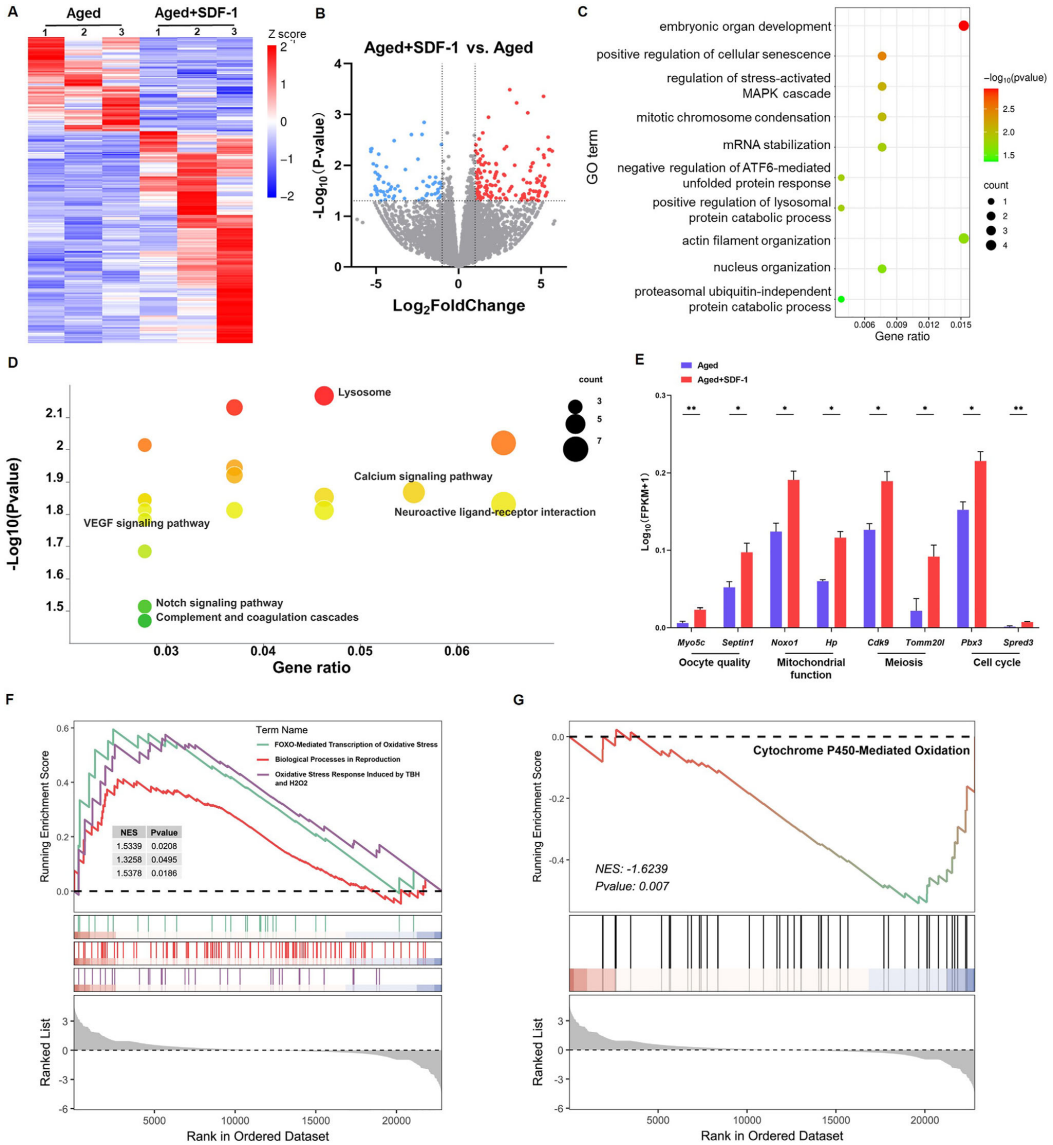
Effects of SDF‐1 supplementation on the transcriptome level of oocytes from aged mice. (A) Heatmap illustration displaying the gene expression profiles in oocytes from aged and aged+SDF‐1 mice. (B) Volcano plot displays DEGs (upregulated, red; downregulated, blue; no difference, grey) in oocytes from aged+SDF‐1 mice compared to those from aged mice. (C) GO analysis (biological process) of DEGs in oocytes from aged+SDF‐1 mice compared to those from aged mice. (D) KEGG analysis of DEGs in oocytes from aged+SDF‐1 mice compared to those from aged mice. (E) RNA‐seq results of selected DEGs in the oocyte quality and development pathway in oocytes from aged+SDF‐1 mice compared to those from aged mice. (F) GSEA expression upregulated trend in oocytes from aged+SDF‐1 mice compared to those from aged mice. (G) GSEA expression downregulated trend in oocytes from aged+SDF‐1 mice compared to those from aged mice. *, *p <* 0.05; **, *p <* 0.01.

To elucidate the mechanisms by which SDF‐1 improves oocyte quality in aged mice, single‐oocyte RNA sequencing was performed on oocytes from aged mice with or without SDF‐1 treatment. Heatmap analysis revealed that transcriptomic profiling of oocytes from aged mice greatly changed after SDF‐1 supplementation (Figure 6A). Volcano plots identified 326 significantly differentially expressed transcripts, including 225 upregulated and 101 downregulated genes, in SDF‐1‐treated oocytes compared with untreated controls (Figure 6B). Gene ontology (GO) analyses indicated that these differentially expressed genes (DEGs) were primarily associated with biological processes related to development, RNA metabolism and translation, proteostasis and stress response, and senescence, such as embryonic organ development, positive regulation of cellular senescence, regulation of stress‐activated MAPK cascade, negative regulation of ATF6‐mediated unfolded protein response, positive regulation of lysosomal protein catabolic process, and proteasomal ubiquitin‐independent protein catabolic process (Figure 6C). Consistent with these findings, KEGG pathway analysis suggested the involvement of lysosome and neurodegeneration‐relevant pathways, including neuroactive ligand‐receptor interaction, complement and coagulation cascades, calcium signaling pathway, VEGF signaling pathway, and Notch signaling pathway (Figure 6D). Moreover, the expression of several genes involved in oocyte quality *(Myo5c*, *Septin1*), mitochondrial function (*Noxo1*, *Hp*), meiosis (*Cdk9*, *Tomm20l*), and cell cycles (*Pbx3*, *Spred3*) was remarkably altered following SDF‐1 treatment (Figure  6E). In addition, Gene Set Enrichment Analysis (GSEA) revealed upregulation of gene sets involved in FOXO‐mediated transcription of oxidative stress, biological processes in reproduction, and oxidative stress response induced by TBH and H2O2, and downregulation of those related to cytochrome P450‐mediated oxidation, suggesting a significant role for oxidative stress in the SDF‐1‐mediated transcriptomic alteration (Figure 6F,G). Taken together, the improvement of oocyte quality from aged mice induced by SDF‐1 supplementation might be remodeling the oocyte transcriptome.

### 2.6 SDF‐1 Ameliorates Oocyte Aging Through Enhanced Autophagy Activation

**FIGURE 7:**
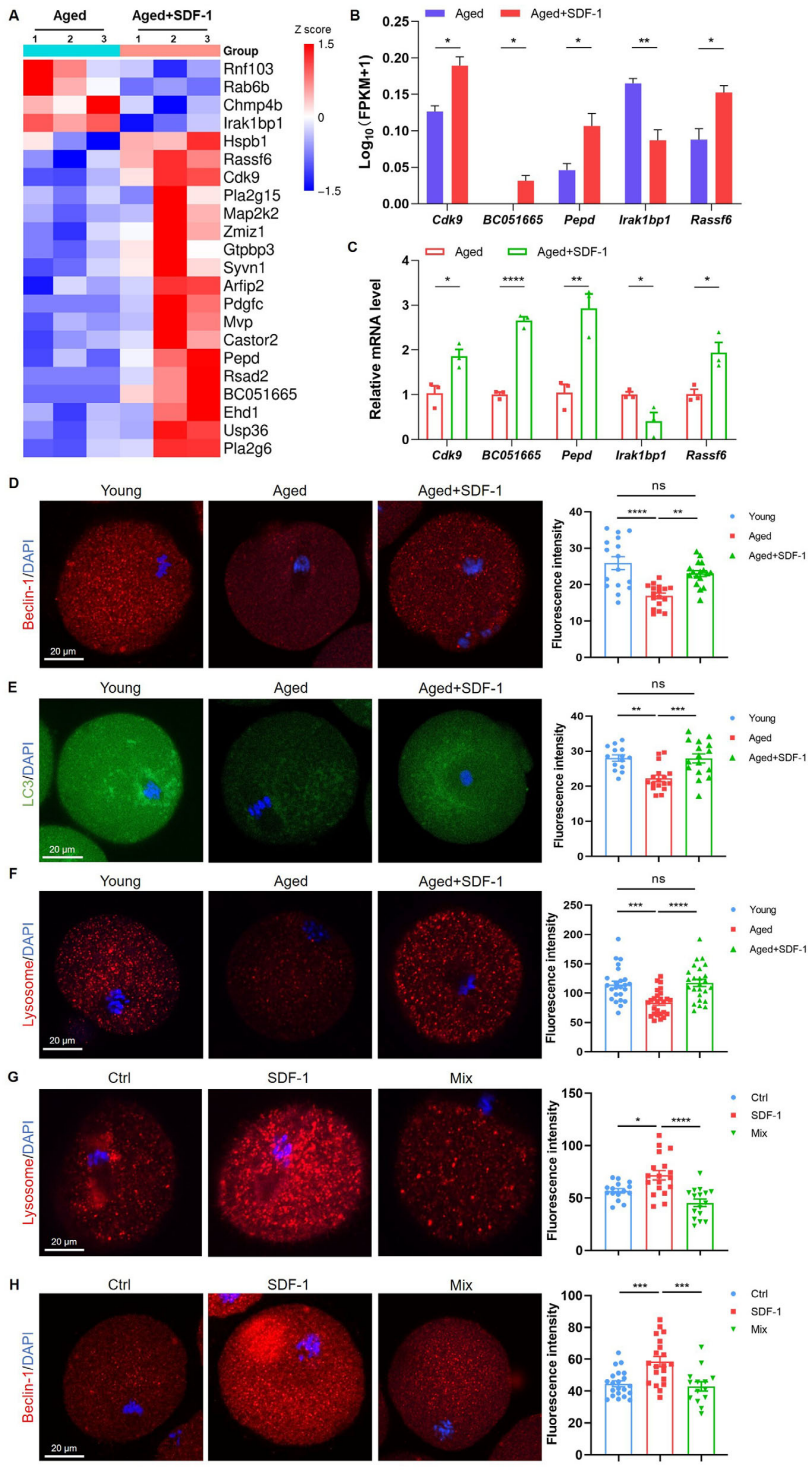
SDF‐1 supplementation induces autophagy modulation in oocytes from aged mice. (A) Heatmap illustration displays DEGs in the autophagy pathway in oocytes from aged and aged+SDF‐1 mice. (B) RNA‐seq results of selected DEGs in the autophagy pathway in oocytes from aged mice compared to those from aged+SDF‐1 mice. (C) Validation of RNA‐seq data in autophagy pathway by quantitative RT‐PCR in oocytes from aged and aged+SDF‐1 mice. (D) Representative images and relative fluorescence intensity of Beclin‐1 in oocytes from young, aged, and aged+SDF‐1 mice. (E) Representative images and relative fluorescence intensity of LC3 in oocytes from young, aged and aged+SDF‐1 mice. (F) Representative images and fluorescence intensity of lysosome in oocytes from young, aged, and aged+SDF‐1 group. (G) Representative images and fluorescence intensity of lysosome in oocytes from control, SDF‐1, and Mix group. (H) Representative images and fluorescence intensity of Beclin‐1 in oocytes from control, SDF‐1 and Mix group. Ctrl, control; Mix, CQ+SDF‐1. *, *p <* 0.05; **, *p <* 0.01; ***, *p <* 0.001; ****, *p <* 0.0001; ns, not significant.

Transcriptomic analysis revealed that SDF‐1 treatment significantly altered the expression of autophagy‐related genes (Figure 7A). This transcriptional profile was corroborated by RT‐PCR, which showed consistent changes in the mRNA levels of these genes, including *Cdk9*, *BC051665*, *Pepd*, *Irak1bp1*, and *Rassf6* (Figure 7B,C). To validate these observations at the protein level, immunofluorescence staining was performed for the autophagosome markers Beclin‐1 and LC3. The intensity of both markers was reduced in oocytes from aged mice but was restored to younger levels following SDF‐1 treatment in vitro (Figure 7D,E). Furthermore, SDF‐1 supplementation enhanced lysosome‐associated fluorescence in oocytes (Figure 7F), suggesting the improvement of autophagy. To determine whether the beneficial effects of SDF‐1 depend on autophagy activation, oocytes from aged mice were treated with SDF‐1 in the presence or absence of CQ, a known autophagy inhibitor. As expected, CQ treatment effectively suppressed lysosomal fluorescence intensity (Figure S4A), Beclin‐1 signal (Figure S4B), and simultaneously led to ROS accumulation (Figure S4C) and mitochondrial abnormalities (Figure S4D,E). Importantly, compared to the SDF‐1 group, co‐treatment with CQ and SDF‐1 (Mix) significantly reduced the intensity of lysosomal signals (Figure 7G) and Beclin‐1 (Figure 7H), indicating that CQ effectively blocked SDF‐1‐mediated autophagy.

### 2.7 Autophagy‐Driven Stress Granule Clearance Plays Central Role in the Improvement of SDF‐1 on Aged Oocytes

**FIGURE 8:**
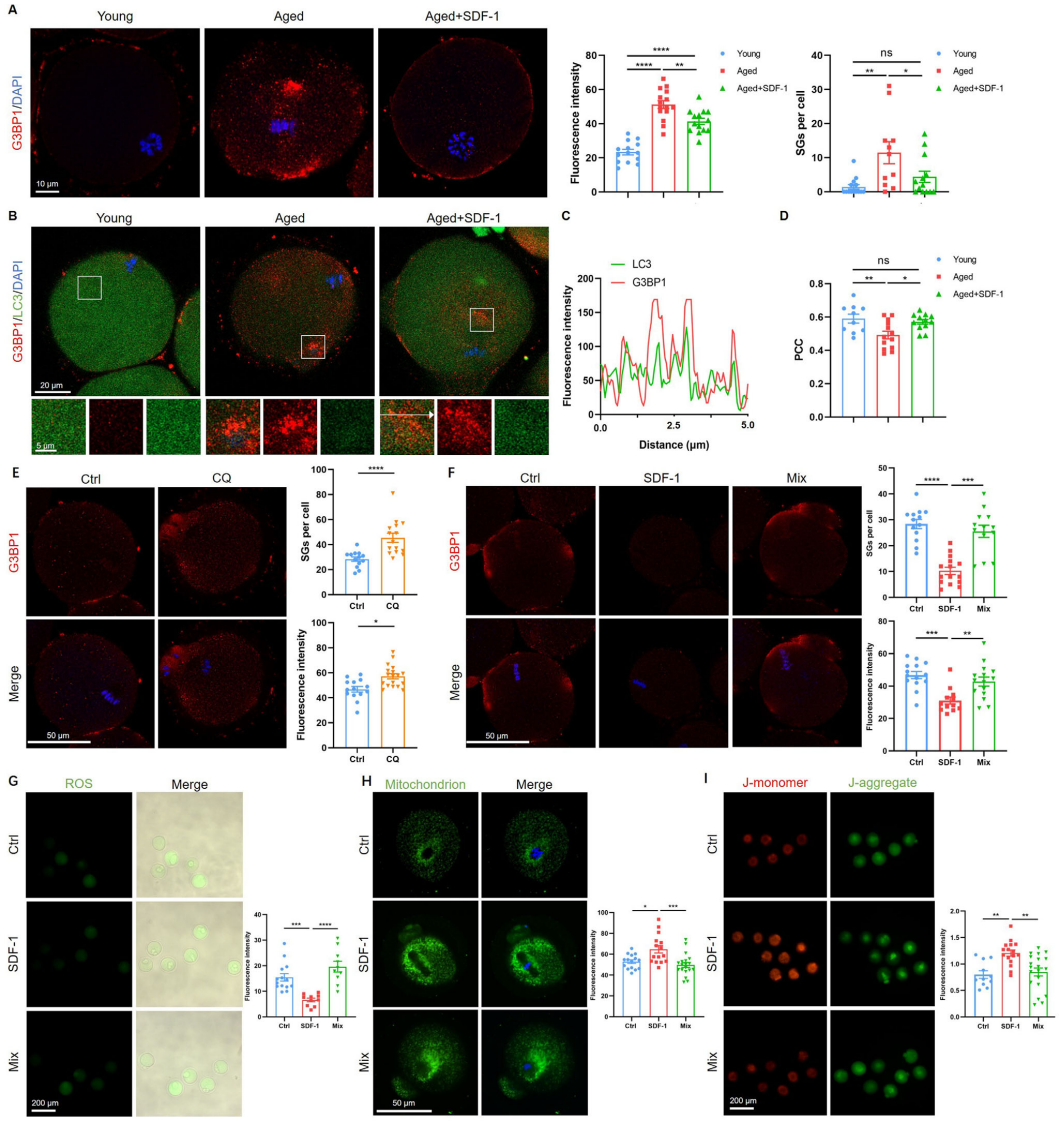
Autophagy‐driven stress granule clearance play central role in the improvement of SDF‐1 on aged oocytes. (A) Representative images and relative fluorescence intensity of G3BP1 and quantification of SGs in oocytes from young, aged, and aged+SDF‐1 mice. (B) Representative images of G3BP1 and LC3 localization in oocytes from young, aged and aged+SDF‐1 group. (C) Fluorescence intensity of G3BP1 and LC3 signals along the indicated white arrow from (B). (D) Pearson correlation coefficient (PCC) analysis of signals from G3BP1 and LC3 in oocytes from young, aged and aged+SDF‐1 group. (E) Representative images and relative fluorescence intensity of G3BP1 and quantification of SGs in oocytes from control and CQ group. (F) Representative images and relative fluorescence intensity of G3BP1 and quantification of SGs in oocytes from control, SDF‐1 and Mix group. (G) Representative images and fluorescence intensity of ROS level in oocytes from control, SDF‐1 and Mix group. (H) Representative images and fluorescence intensity of mitochondrion staining by mito‐tracker in oocytes from control, SDF‐1, and Mix group. (I) Representative images and fluorescence intensity of mitochondrial membrane potential as assessed by JC‐1 staining in oocytes from control, SDF‐1 and Mix group. Ctrl, control; CQ, chloroquine; Mix, CQ+SDF‐1; SG, stress granule. *, *p <* 0.05; **, *p <* 0.01; ***, *p <* 0.001; ****, *p <* 0.0001; ns, not significant.

Stress granules are transient membraneless structures that form during oxidative stress to relieve stress damage and promote cell survival. Consistent with its role as a stress granule biomarker, the fluorescence intensity of G3BP1 was significantly reduced following SDF‐1 treatment, demonstrating effective clearance of stress granules in oocytes (Figure 8A). Interestingly, it was found that the expression of G3BP1 was colocalized with LC3 (Figure 8B,C), while the correlation between LC3 and G3BP1 fluorescence signals was markedly weaker in oocytes from aged mice compared to those treated with SDF‐1 by PCC analyses (Figure 8D). This strengthened association after treatment suggests that SDF‐1‑enhanced autophagy is closely linked to stress granule clearance in aged oocytes. Moreover, CQ supplementary enhanced the expression of G3BP1, which suggested that inhibition of autophagy retarded scavenging the stress granules in oocytes (Figure 8E). Furthermore, the suppression of autophagy by CQ abolished the protective effects of SDF‐1 on intracellular stress granule clearance (Figure 8F), resulting in abnormal ROS accumulation (Figure 8G), and an abnormal clustered in mitochondrial pattern (Figure 8H), as well as decreased mitochondrial membrane potential (Figure 8I) in the Mix group. Therefore, these results demonstrated that SDF‐1 ameliorates oocyte aging mechanistically through the promotion of autophagy activation‐mediated stress granule scavenging.

## 3 Discussion

The current study has revealed a novel role of SDF‐1 in counteracting maternal age‐related decline in oocyte quality. Systematic analysis across human and murine samples revealed lower SDF‐1 levels in aged reproductive tissues, including follicle fluid, oocytes, and ovaries, establishing its potential as a biomarker of reproductive aging. In both in vitro and in vivo supplementation experiments, SDF‐1 ameliorated multiple aging‐associated defects, such as spindle abnormality, chromosome disarrangement, organelle mislocalization, and elevated oxidative stress, leading to improved oocyte quality and restored fertility in aged mice. Transcriptomic and molecular analyses further indicated that SDF‐1 may enhance autophagic activity, thereby facilitating ROS clearance and the elimination of persistent stress granules. These observations support a mechanistic pathway through which SDF‑1 alleviates aged‐induced oocyte deterioration.

SDF‐1, a cytokine known to signal primarily through CXCR4, has been implicated in female fertility and embryonic development [14, 18]. Our data extend these observations by revealing an inverse correlation between SDF‐1 levels and ovarian aging in both human and mice, suggesting a conserved function across species. In clinical samples, SDF‐1 concentrations in follicular fluid correlated positively with AFC, a key marker of ovarian reserve [19]. In aged mice, SDF‐1 supplementation effectively attenuated age‐related declines in ovulation rates, reduced oocyte morphological abnormalities, and suppressed follicular atresia, accompanied by increased litter size. Collectively, these findings raise the possibility that SDF‑1 may serve as a useful indicator of ovarian reserve and oocyte quality, although further validation in larger clinical cohorts is required to establish its utility in predicting reproductive outcomes.

Using both in vivo and in vitro approaches, we observed that SDF‐1 supplementation improved several parameters of oocyte quality in aged mice. Oocyte quality assessment typically encompasses nuclear and cytoplasmic markers [20]. In MII oocytes, nuclear competence is reflected by proper spindle morphology and chromosome alignment [21]. In accordance with previous studies on other supplementation agents [22], our results showed that SDF‐1 treatment reduced the incidence of spindle abnormalities and chromosomal misalignment, suggesting a beneficial effect on nuclear maturation. With respect to cytoplasmic quality, which relies heavily on organelle distribution and function [23], we found that SDF‑1 treatment improved mitochondrial distribution and membrane potential, and promoted a more physiological CGs distribution [22], all of which are associated with enhanced fertilization capacity and subsequent embryonic development. These observations are consistent with earlier findings in porcine and ovine models, where SDF‐1 supplementation during in vitro maturation improved oocyte competence [15, 22]. In summary, our data suggest that SDF‐1 may have therapeutic potential for improving the quality of ageing oocytes, although further studies are needed to confirm its efficacy and safety in clinical settings.

To explore the mechanisms underlying SDF‐1‑mediated improvement in oocyte quality, we performed single‐oocyte transcriptomic profiling on aged mouse oocytes with or without SDF‐1 treatment. The results pointed to a potential involvement of lysosome‐dependent autophagic processes. Specifically, SDF‐1 treatment was associated with upregulated expression of autophagy‐related markers, including Beclin‐1 and LC3, and increased lysosomal activity. Pharmacological blockade of autophagy using CQ largely abrogated the beneficial effects of SDF‐1 on mitochondrial function, stress granule clearance, and oocyte maturation rates. However, because CQ acts by inhibiting autophagosome‐lysosome fusion and may broadly affect lysosomal function, these data indicate that lysosome‐dependent quality‐control pathways are likely involved, but they do not establish canonical autophagy as the exclusive mediator. Further studies using genetic approaches, such as conditional knockout of core autophagy genes, will be required to delineate the precise contribution of autophagy to the SDF‑1‑mediated rescue of aged oocytes.

Our findings suggest that autophagy may serve as a downstream effector of SDF‐1 in aged oocytes. Autophagy, and particularly mitophagy, is well recognized for its role in clearing oxidative metabolites and preserving organelle homeostasis [24]. In our study, SDF‐1 treatment reduced ROS and superoxide levels, increased GSH content, and restored mitochondrial membrane potential. These observations are consistent with the notion that enhanced autophagy could contribute to oocyte quality improvement, potentially through the removal of dysfunctional mitochondria and alleviation of oxidative damage, thereby reducing DNA double‑strand breaks and apoptosis. This interpretation aligns with previous studies emphasizing the importance of redox homeostasis in oocyte competence [25]. Nevertheless, given the correlative nature of these data, direct causal links between SDF‑1‑induced autophagy and the observed redox changes require further experimental confirmation.

Stress granules are cytoplasmic ribonucleoprotein condensates that assemble in response to cellular stress and have been implicated in various age‐related pathologies [26]. Their accumulation is considered a marker of impaired proteostasis and diminished adaptive stress capacity [27], both of which are hallmarks of aging. Recent studies have linked persistent stress granules to ovarian aging, and their clearance has been proposed as a protective mechanism against age‐related ovarian dysfunction [28]. Autophagy, a lysosomal degradation pathway that removes aggregated proteins and damaged organelles [29], has been suggested to participate in stress granule homeostasis [27], although evidence in oocytes remains limited. In our study, aged oocytes exhibited increased levels of the stress granule marker G3BP1, and SDF‐1 treatment was associated with enhanced autophagic activity and reduced G3BP1 signal. Inhibition of autophagy attenuated both stress granule clearance and the quality‑improving effects of SDF‑1, suggesting a functional interplay between these processes. These observations align with the established role of stress granules in cellular stress response [30, 31], and corroborate their documented association with ovarian aging [28]. Collectively, our findings point to persistent stress granules as a potential contributor to oocyte senescence and raise the possibility that the autophagy‑stress granule clearance axis may represent a previously underexplored pathway for therapeutic intervention in reproductive aging. However, further studies are needed to confirm the causal relationship and translational relevance of these observations.

Our findings suggest that SDF‑1 supplementation may help counteract the decline in oocyte quality associated with reproductive aging, offering preliminary evidence to support further exploration of its translational potential. The data highlight SDF‑1 as a candidate anti‑aging factor and indicate that enhanced autophagy may serve as one mechanism underlying its effects on cellular quality control. From a translational standpoint, SDF‑1 or its stable analogues could conceivably be developed as supplements to improve oocyte quality in older IVF patients, and ovarian‑targeted delivery strategies may represent a future avenue for intervention [32]. However, given the current paucity of research in this area, particularly regarding safety, optimal dosing, and long‑term efficacy, these possibilities remain speculative and will require thorough preclinical and clinical investigation before any clinical application can be considered.

However, this study had several limitations. Firstly, our results originated from mouse models and the efficacy and safety of SDF‐1 supplementation in humans require rigorous validation. Secondly, while we have confirmed that enhanced lysosome‐dependent autophagy is a key downstream mechanism underlying the anti‐aging effects of SDF‐1, the precise upstream receptor‐dependent signaling cascades, including CXCR4‐ or CXCR7‐ mediated ligand engagement and the subsequent activation of the AMPK–mTORC1 or MAPK axes, that connect SDF‑1 stimulation to the initiation of autophagy remain incompletely characterized in our current model. Consequently, further mechanistic studies employing targeted gene‑knockout mouse models or selective receptor antagonists are warranted to rigorously dissect the molecular interface between SDF‑1 receptor signaling and the autophagic clearance of stress granules in aged oocytes. Furthermore, the long‐term effects of SDF‐1 supplementation in humans, as well as the optimal dosage and timing of administration, remain unclear. Comprehensive, long‐term, in‐depth in vivo studies in primates and humans are required in the future.

## 4 Conclusion

In summary, our findings suggest a role for SDF‐1 in age‐related ovarian ageing and indicate that SDF‐1 supplementation may improve oocyte quality and fertility in aged mice. This effect appears to be associated with enhanced autophagy‐mediated scavenging of stress granules, although the precise upstream signaling pathways and the causal relationship between autophagy and stress granule removal remain to be fully defined. Overall, these observations position SDF‐1 as a candidate worthy of further investigation for its potential to extend female reproductive lifespan and improve reproductive outcomes in advanced‑age women.

## 5 Methods

### 5.1 Clinical Specimen Collection

Follicular fluid and immature oocytes were obtained from patients undergoing IVF at the Reproductive Medicine Center, Tongji Hospital, Tongji Medical College, Huazhong University of Science and Technology (Wuhan, China). All participants provided informed consent for the donation of these materials for research purposes, and this study was approved by the Ethics Committee of Tongji Hospital, Tongji Medical College, Huazhong University of Science and Technology (TJ‐IRB202410032). Patients diagnosed with tubal or male factor infertility were included. Exclusion criteria consisted of endometriosis, adenomyosis, tumor, chromosomal abnormalities, or other complications. Controlled ovarian hyperstimulation (COH) was carried out following standardized protocols, as described previously [33]. The clinical characteristics of enrolled patients were obtained from the record system of our center and are summarized in Table S1.

Following follicular aspiration, the follicular fluid was examined under a stereomicroscope to identify and retrieve cumulus‐oocyte complexes (COCs). After denudation, the immature oocytes were collected and fixed in 4% paraformaldehyde for subsequent fluorescence staining. The retained follicular fluid was collected and centrifuged at 1500 × *g* for 20 min. The resulting follicular supernatant was then transferred into tubes and stored at ‐80°C for future SDF‐1 concentration detection.

### 5.2 Animals

All animal protocols and experiments were approved by the Ethical Committee of Tongji Hospital, Tongji Medical College, Huazhong University of Science and Technology (TJH‐202312019). Young (6‐8‐week‐old) and reproductively aged (44‐48‐week‐old) ICR female mice were kept at an appropriate temperature (23°C–25°C) and illumination (12 h light‐dark cycle), and had free access to food and water. Recombinant mouse SDF‐1 protein (Abclonal, RP01877) was delivered intraperitoneally to the animals in the aged+SDF‐1 group at a dose of 10 µg/kg body weight per day according to a previous study [34]; the animals in the young and aged groups received the same amount of vehicle. The injections were administered for 14 consecutive days. Then, a subset of female mice were humanely euthanized by cervical dislocation. Mice and ovaries were weighed separately to calculate the ovarian index [ovarian index (%) = weight of one ovary/body weight × 100%]. Young, aged, and aged+SDF‐1 group female mice (n = 6 for each group) were co‐housed continuously with fertile ICR male mice (10‐week‐old) for 14 weeks. The number of litters and pups was recorded for fertility analysis. The number of pups per female was defined as the cumulative mean offspring produced per mouse over the 14‐week period, while the number of pups per litter referred to the mean offspring count for each individual litter.

### 5.3 Mice Oocyte Collection and Culture

Young and aged female ICR mice were intraperitoneally injected with 10 IU pregnant mare serum gonadotropin (PMSG) (Solarbio, P9970) and sacrificed by cervical dislocation after 48 h. Bilateral ovaries were immediately excised and placed in the M2 medium (Aibei, M1250) containing 50 µM isobutylmethylxanthine (IBMX) (Selleck, S5836). The oocytes were released via puncturing the ovaries with sterile needles, then thoroughly washed several times with M2 medium to remove cumulus cell debris and impurities. GV oocytes, characterized by the presence of a visible nucleus (germinal vesicle) within the cytoplasm, were collected and cultured in vitro in (1) M2 medium; (2) M2 medium with SDF‐1 (Abclonal, RP01877); (3) M2 medium with 50 µM chloroquine (CQ) (MedChemExpress, HY‐17589A; (4) M2 medium with SDF‐1 and 50 µM CQ for 16 h at 37°C with 5% CO_2_. Two hours later, the number of MI oocytes undergone germinal vesicle breakdown (GVBD) were recorded and 16 h later, MII oocytes were verified with the extrusion of the polar body 1 (PB1). For collection of ovulated oocytes, female mice were intraperitoneally injected with 10 IU PMSG (Solarbio, P9970), followed by a subsequent injection of 10 IU hCG (Solarbio, YZ‐1817) administered 48 h later. After 14‐16 h, COCs were obtained from oviductal ampullae and exposed to 1 mg/mL hyaluronidase (Aibei, M2215) to isolate the granulosa cells. The number of oocytes, PB1 extrusion rate and fragmentation rate were recorded to evaluate ovulation efficiency.

### 5.4 In Vitro Fertilization and Mice Embryo Culture

All the mediums were prepared and equilibrated for 12 h before the experiment began. The male mice aged 10 weeks were euthanized, and the epididymides were cut out by scissors and forceps. Then we incised the duct of the epididymis, collected pellets of sperm with a dissecting needle, and applied to drops of human tubal fluid (HTF) (Cosmo, KYD‐008‐02EX) on the dish. The dish was incubated at 37°C under an atmosphere of 5% CO_2_, 5% O_2_, and 90% N_2_ for 50 min. Then, a 5 µL sperm suspension was added to a drop of 60 µL HTF medium containing matured oocytes. After fertilization for 4‐6 h, redundant sperm on the zona pellucida (ZP) in the HTF medium were removed by gentle pipetting, and the fertilized oocytes were transferred to the Potassium Simplex Optimized Medium (KSOM) (Cosmo, CSR‐R‐B074) and cultured under an atmosphere of 5% CO_2_, 5% O_2_, and 90% N_2_ at 37°C. The number of 2‐cell stage embryos, cleavage stage embryos and blastocysts were calculated after 24, 48, and 108 h from insemination, respectively.

### 5.5 Sperm‐Binding Assay

In the process of IVF, sperm and oocytes were co‐incubated for 1 h under an atmosphere of 5% CO_2_, 5% O_2_, and 90% N_2_ at 37°C. Following incubation, the oocytes were washed twice and transferred to 4% paraformaldehyde (Servicebio, G1101) for 30 min. Then, the oocytes were stained with DAPI (Servicebio, G1012) for 15 min. The number of spermatozoa tightly bound to the ZP of each oocyte was counted under a Zeiss LSM 900 confocal microscope.

### 5.6 Enzyme‐Linked Immunosorbent Assay (ELISA)

The concentration of SDF‐1 in the human follicular fluid was measured via ELISA kit (Jingmei, JM‐05313H2), following the manufacturer's guideline, and measured using a microplate reader (Labsystems Multiskan MS, Finland) at 450 nm.

### 5.7 Histological Analysis

Mice ovaries were fixed in 4% paraformaldehyde (Servicebio, G1101) for 24 h, then dehydrated through a graded series of ethanol, cleared in xylene, and infiltrated with and embedded in paraffin wax. Serial sections were cut at a thickness of 5 µm using a microtome and mounted onto glass slides. For histological examination, the sections were deparaffinized in xylene and rehydrated through a descending ethanol series to distilled water. They were then stained with Hematoxylin and Eosin (H&E) according to standard protocols. The follicles at different stages were recorded under a light microscope. Primordial, primary, secondary, pre‐antral and antral follicles were defined as previously described [35].

### 5.8 Real‐Time Quantitative PCR

To detect mRNA levels of mice ovaries and oocytes, total mRNA was extracted via NucleoZol reagent (Macherey‐Nagel, Germany) according to the manufacturer's instructions. The cDNA was synthesized with the HiScript II Q RT SuperMix for qPCR (+gDNA wiper) (Vazyme, R223‐01), and the real‐ time PCR reaction was conducted as previous reported (Vazyme, Q711‐02/03) [36]. The primer pairs for RT‐PCR were listed in Table S2. PCR specificity was verified by analyzing melting curve data and gene expression levels were normalized to the internal reference gene GAPDH.

### 5.9 Protein Extraction and Western Blot

The protein of mice ovaries was extracted via RIPA Lysis Buffer (Servicebio, G2002), supplemented with complete protease inhibitor (Servicebio, G2006 and G2007). The samples were homogenized and centrifuged at 12 000 × *g* for 15 min at 4°C. Then appropriate volume of loading buffer was added to the supernatant and the mixture was heated at 95°C for 5 min for subsequent western blotting. The proteins samples were separated using 4%–20% polyacrylamide gels (ACE, ET10420) following the manufacturer's instructions. Then we transferred the protein bands from gels to the polyvinylidene fluoride (PVDF) membranes (GE Healthcare Life Sciences, 10600023) and blocked in 5% skim milk (Servicebio, GC310001) for 1 h. Membranes were incubated with anti‐rabbit CXCL12 antibody (1:1000, Abclonal, A1325) overnight at 4°C and with HRP conjugated goat anti‐rabbit IgG (Servicebio, GB23303) for 1 h at room temperature. The immunoreactive bands were detected using SuperFemto ECL Chemiluminescence Kit (Vazyme, E423) with Tanon imaging System. The relative protein levels were normalized to GAPDH to standardize the loading control.

### 5.10 Immunofluorescent Staining

Oocytes were fixed in 4% paraformaldehyde (Servicebio, G1101) for 30 min. Then, the cells membranes were permeabilized and blocked with the mixture of 3% bovine serum albumin (BSA) (Servicebio, GC305010) and 0.1% Triton X‐100 (Servicebio, GC204003) for 1 h. Oocytes were washed three times with 1% BSA and incubated overnight at 4°C with primary antibodies (anti‐rabbit CXCL12 antibody, 1:200, Abclonal, A1325; anti‐mouse β‐tubulin antibody, 1:100, Cell Signaling, 86298; anti‐rabbit phospho‐histone H2AX antibody, 1:200, Cell Signaling, 9718; anti‐rabbit LC3A/B antibody, 1:200, Cell Signaling, 12741; anti‐rabbit Beclin‐1 antibody, 1:200, Abcam, AB207612; anti‐mouse G3BP1 antibody, 1:100, Proteintech, 66486). The next day, the oocytes were washed three times with 1% BSA and incubated for 1 h in dark with secondary antibodies (goat anti‐rabbit IgG labeled with cyanine 3, 1:200, Servicebio, GB22301; goat anti‐rabbit IgG labeled with FITC, 1:200, Servicebio, GB22303). Then, the oocytes were stained with DAPI (Servicebio, G1012) for 15 min. The stained oocytes were mounted on the confocal dishes and examined via Zeiss LSM 900 confocal microscope. Colocalization was measured with the Pearson correlation coefficient (PCC) by ImageJ software. PCC values ranged from 1 for positive correlation of two images to −1 for negative correlation of two images.

### 5.11 Cortical Granule (CGs) Staining

As described before, oocytes fixed with 4% paraformaldehyde (Servicebio, G1101) for 30 min. 3% BSA (Servicebio, GC305010) and 0.1% Triton X‐100 (Servicebio, GC204003) were used to permeabilize and block the oocytes. Then, oocytes were washed three times with 1% BSA and incubated with 20 µg/mL FITC‐labeled lens culinaris agglutinin (LCA) (Invitrogen, L32475) for 1 h in 37°C. Finally, the oocytes were stained with DAPI (Servicebio, G1012) for 15 min and mounted on the confocal dishes. Zeiss LSM 900 confocal microscope was used to observed the CGs. Normally, in matured MII oocyte, CGs achieve a definitive, uniform monolayer lining directly beneath the plasma membrane. Meanwhile, CGs absent from the region overlying the meiotic spindle, which is typically located adjacent to the polar body.

### 5.12 Mitochondrial Function Assessment

To evaluate mitochondrial function, matured oocytes were incubated in M2 medium with 500 nM Mito‐tracker Green (Beyotime, C1048) for 30 min at 37°C with 5% CO_2_. After that, oocytes were stained with Hoechst 33342 (Servicebio, G1127) for 15 min and washed with M2 medium for 3 times. Then, the oocytes were mounted on the confocal dishes and captured by Zeiss LSM 900 confocal microscope.

Mitochondrial membrane potential was assessed using the Mitochondrial Membrane Potential Assay Kit with JC‐1 (Beyotime, C2006). Oocytes were incubated in a mixture of 50 µL M2 medium and 50 µL JC‐1 dye working solution at 37°C for 30 min in dark. Following incubation, the oocytes were washed three times with JC‐1 staining buffer and immediately observed under a fluorescence microscope (Axio Observer A1; Carl Zeiss, Germany). At high mitochondrial membrane potential, red fluorescence was observed, while green fluorescence was noted at low mitochondrial membrane potential. Mitochondrial membrane potential changes were quantified using image analysis software by calculating the ratio of aggregated JC‐1 (red fluorescence) to monomeric JC‐1 (green fluorescence). A decrease in the red‐to‐green fluorescence ratio indicates mitochondrial depolarization.

### 5.13 Determination of Oxidative Stress Level

Total reactive oxygen species (ROS) levels and mitochondrial ROS levels in oocytes were assessed by the ROS‐specific fluorescent probe 2’,7’‐dichlorodihydrofluorescein diacetate (DCFH‐DA) (Beyotime, S0033S) and Mito SOX Red (Beyotime, S0061S), respectively. DCFH‐DA and Mito SOX were diluted to a final concentration of 10 µM and 5 µM in M2 medium, respectively. The oocytes were incubated at 37°C for 30 min in the dark. Following three washes with M2 medium, ROS levels were immediately observed under a fluorescence microscope (Axio Observer A1; Carl Zeiss, Germany). In order to measure the level of glutathione (GSH), monochlorobimane (MedChemExpress, HY‐101899) was diluted to a final concentration of 10 µM in M2 medium. Matured oocytes were incubated at 37°C for 30 min in the dark. Following three washes with M2 medium, GSH levels were observed by Zeiss LSM 900 confocal microscope.

### 5.14 Annexin V Staining

Following the removal of the ZP via acidic Tyrode's solution (LEAGENE, CZ0060), oocytes were washed thoroughly in binding buffer and incubated in the dark for 15 min at room temperature in a droplet of Annexin V‐FITC (1:100, Servicebio, G1511). After incubation, oocytes were washed gently in the binding buffer to remove unbound dye. Stained oocytes were immediately examined under Zeiss LSM 900 confocal microscope.

### 5.15 RNA Sequencing and Analysis

Transcriptomic analysis of MII oocytes derived from in vitro maturation was carried out using a protocol for SMART‐Seq. In brief, 3 samples were collected for the aged and aged+SDF‐1 group (15 oocytes per sample) in the lysis buffer that contains RNase inhibitors. Oligo dT was used to directly reverse‐transcribed mRNA. The reverse‐transcribed cDNA was amplified through PCR and purified to construct a library. The constructed libraries were sequenced on an Illumina platform with a 150 bp paired‐end strategy. The raw sequencing data were processed to remove low‐quality bases and adapter sequences, yielding clean reads that served as the basis for all downstream analyses.

### 5.16 Lysosome Function Assessment

Matured oocytes were incubated in M2 medium with Lyso‐Tracker Red (1:1000, Beyotime, C1046) for 30 min at 37°C with 5% CO_2_. Then, Hoechst 33342 (Servicebio, G1127) was used to stain cell nucleus for 15 min, and captured by Zeiss LSM 900 confocal microscope.

### 5.17 Statistical Analysis

The experiments were performed with three independent biological replicates. All the data were analyzed via SPSS software 22.0 and presented by GraphPad Prism 8.0. All continuous data with normal distribution were confirmed via Student's t‐test and expressed as mean ± SEM; otherwise, the Kruskal–Wallis nonparametric method was performed for non‐normal distributed data and presented as the median (interquartile range [IQR]). Moreover, the categorical data are shown as the number of cases and frequency (percentage), with Chi‐square test. Statistical significance was defined as a two‐tailed *p* value <0.05.

## Author Contributions

**Rui Long**: conceptualization, methodology, validation, visualization, writing – original draft, project administration, software. **Meng Wang**: funding acquisition, writing – original draft, writing – review and editing, resources, supervision, validation, formal analysis. **Ruolin Mao**: methodology, formal analysis, validation. **Hong Chen**: validation, visualization, software. **Juepu Zhou**: validation, data curation, investigation. **Xiangfei Wang**: data curation, project administration. **Zheng Yuan**: methodology. **Ri‐Cheng Chian**: conceptualization, methodology. **Nan Xiao**: funding acquisition, resources, supervision, project administration. **Qingsong Xi**: investigation, data curation, supervision, methodology, writing – review and editing. **Yimin Shu**: methodology, conceptualization, writing – review and editing. **Lei Jin**: conceptualization, methodology, data curation, funding acquisition, writing – review and editing. **Lixia Zhu**: conceptualization, methodology, writing – review and editing, project administration, data curation.

## Funding

This study was supported by the National Key Research and Development Program of China (2025YFC3508002 and 2024YFC2706700), National Natural Science Foundation of China (82401948) and Tianjin Health Research Project (TJWJ2023MS028).

## Conflicts of Interest

The authors declare no conflicts of interest.

## Supporting information




**Supporting File 1**: advs76902‐sup‐0001‐SuppMat.docx.

**Supporting File 2**: advs76902‐sup‐0002‐FigureS1‐S4.zip.

## Data Availability

The raw data have been deposited in the Genome Sequence Archive (GSA: CRA037570). All other data supporting the findings of this study are available from the corresponding author upon reasonable request. This study did not involve human clinical trials and was therefore not registered in a clinical trial registry.
