# Supplementary material for: SDF‐1 Attenuates Oocyte Quality Decline During Reproductive Aging Through Autophagy‐Enhanced Stress Granule Scavenging
Source: Adv Sci (Weinh). 2026 Aug 3;13(59):e76902. doi: 10.1002/advs.76902 (PMC13430927; doi:10.1002/advs.76902)
Supplement: Supplementary file 1 [file ADVS-13-e76902-s001.docx]

**Supplemental Figure 1 SDF-1 supplementation promotes *in vitro* maturation of oocytes from aged mice.**

(A) Representative images of *in vitro* cultured oocytes with time from young, aged and aged+SDF-1 group. (B) The percentage of GVBD and PB1 extrusion in oocytes from young, aged and aged+SDF-1 mice after *in vitro* maturation. GVBD, germinal vesicle breaks down; PB, polar body. **, *P*<0.01; ns, not significant.

**Supplemental Figure 2 The *in vivo* supplementation of SDF-1 improves the fertilization capacity and embryo development potential of oocytes from aged mice.**

(A) Representative images of sperm binding to the ZP surrounding oocytes and the number of sperm binding to the ZP of oocytes from young, aged and aged+SDF-1 mice. (B) Representative images of embryos and the fertilization index (the fertilization rate, cleavage rate and blastocyst formation rate) in young, aged and aged+SDF-1 group. ZP, zona pellucida. *, *P*<0.05; **, *P*<0.01; ***, *P*<0.001; ****, *P*<0.0001; ns, not significant.

**Supplemental Figure 3 The *in vivo* supplementation of SDF-1 decreases the oxidative stress level in aged mice MII oocytes.**

(A) Representative images and fluorescence intensity of ROS levels in oocytes at metaphase II from young, aged and aged+SDF-1 group. (B) Representative images and fluorescence intensity of superoxide levels as detected by Mito Sox staining in oocytes at MII from young, aged and aged+SDF-1 group. *, *P*<0.05; **, *P*<0.01; ****, *P*<0.0001; ns, not significant.

**Supplemental Figure 4 Effects of autophagy inhibition on the aged oocytes.**

(A) Representative images and fluorescence intensity of lysosome in oocytes from control and CQ group. (B) Representative images and fluorescence intensity of Beclin-1 in oocytes from control and CQ group. (C) Representative images and fluorescence intensity of ROS level in oocytes from control and CQ group. (D) Representative images and fluorescence intensity of mitochondrion staining by mito-tracker in oocytes from control and CQ group. (E) Representative images and fluorescence intensity of mitochondrial membrane potential as assessed by JC-1 staining in oocytes from control and CQ group. Ctrl, control; CQ, chloroquine. ***, *P*<0.001; ****, *P*<0.0001; ns, not significant.

**Supplemental Table 1 Baseline characteristics of young and aged groups**

|  | Young  (n=105) | Aged  (n=77) | *P* value |
| --- | --- | --- | --- |
| Age, years | 30 (28-31) | 40 (37-42) | **<0.001** |
| Female BMI, kg/m^2^ | 21.5 (20.2-23.5) | 22.9 (20.8-24.4) | 0.108 |
| Female basal FSH, mIU/mL | 8.0 (6.5-10.5) | 9.7（7.8-12.4) | **0.006** |
| Antral follicle count, n | 7 (4-13) | 4 (3-5) | **<0.001** |
| Female AMH, ng/mL | 1.4 (0.8-4.8) | 0.9 (0.4-1.2) | **<0.001** |
| Type of infertility, % (n/N) |  |  | **0.003** |
| Primary | 73.3 (77/105) | 51.9 (40/77) |  |
| Secondary | 26.7 (28/105) | 48.1 (37/77) |  |
| Duration of infertility, years | 2 (1-3) | 2 (1-4) | 0.814 |
| Infertility cause, % (n/N) |  |  | 0.236 |
| Female factor | 58.1 (61/105) | 68.8 (53/77) |  |
| Male factor | 23.8 (25/105) | 14.3 (11/77) |  |
| Bilateral Factor | 18.1 (19/105) | 16.9 (13/77) |  |

**Supplemental Table 2 Primers for qRT-PCR**

| Gene | Primer | Sequence |
| --- | --- | --- |
| *Sdf-1* | Forward | TGCATCAGTGACGGTAAACCA |
|  | Reverse | TTCTTCAGCCGTGCAACAATC |
| *Foxo3* | Forward | TGTCCTATGCCGACCTGA |
|  | Reverse | GTGCCGGATGGAGTTCTT |
| *Mapk* | Forward | ATGGTTTGCTCTGCTTAT |
|  | Reverse | TGATGCCAATGATGTTCT |
| *Sod1* | Forward | TGACTGCTGGAAAGGACG |
|  | Reverse | GCCAATGATGGAATGCTC |
| *Gpx4* | Forward | GATGGAGCCCATTCCTGAACC |
|  | Reverse | CCCTGTACTTATCCAGGCAGA |
| *Nrf2* | Forward | CAGTGCTCCTATGCGTGAA |
|  | Reverse | AGCGGCTTGAATGTTTGT |
| *Cdk9* | Forward | AGAAGCTAGGGTTTGGTCAGC |
|  | Reverse | CACTTGCCAACACCAAGCAC |
| *BC051665* | Forward | TATGCCATCTACCCCACCGT |
|  | Reverse | TGACTCAGCTACCATTCCCCA |
| *Pepd* | Forward | GTCCACTGTGCGGATCCATT |
|  | Reverse | AACACTCGGCATTCCACGAT |
| *Irak1bp1* | Forward | AGGGGTTTCAGGTCTGCATT |
|  | Reverse | TTGCGCCATGCATTCTCAAC |
| *Rassf6* | Forward | CTCAGTCCACACACAGGCAC |
|  | Reverse | AATCAGCTGTCCGTCTTCGG |
| *Gapdh* | Forward | ATTCAACGGCACAGTCAA |
|  | Reverse | TTAGTGGGGTCTCGCTCC |

**Supplemental Table 3 GVBD rates and PB1 extrusion rates of mice oocytes cultured with different concentrations of SDF-1**

| Group | Total No. of oocytes | GVBD rate, % | PB1 extrusion rates, % |
| --- | --- | --- | --- |
| 0 ng/mL SDF-1 | 117 | 87.6±3.4 | 70.3±1.4 |
| 10 ng/mL SDF-1 | 98 | 85.7±2.6 | 74.5±0.4 |
| 20 ng/mL SDF-1 | 93 | 90.0±2.7 | 89.5±0.8^***^ |
| 50 ng/mL SDF-1 | 91 | 84.4±2.8 | 72.2±1.7 |
| 100 ng/mL SDF-1 | 100 | 82.8±0.7 | 71.0±0.4 |

***, *P*<0.001
