# Supplementary figures and images for: SDF‐1 Attenuates Oocyte Quality Decline During Reproductive Aging Through Autophagy‐Enhanced Stress Granule Scavenging
Source: Adv Sci (Weinh). 2026 Aug 3:e76902. Online ahead of print. doi: 10.1002/advs.76902 (PMC13430927; doi:10.1002/advs.76902)

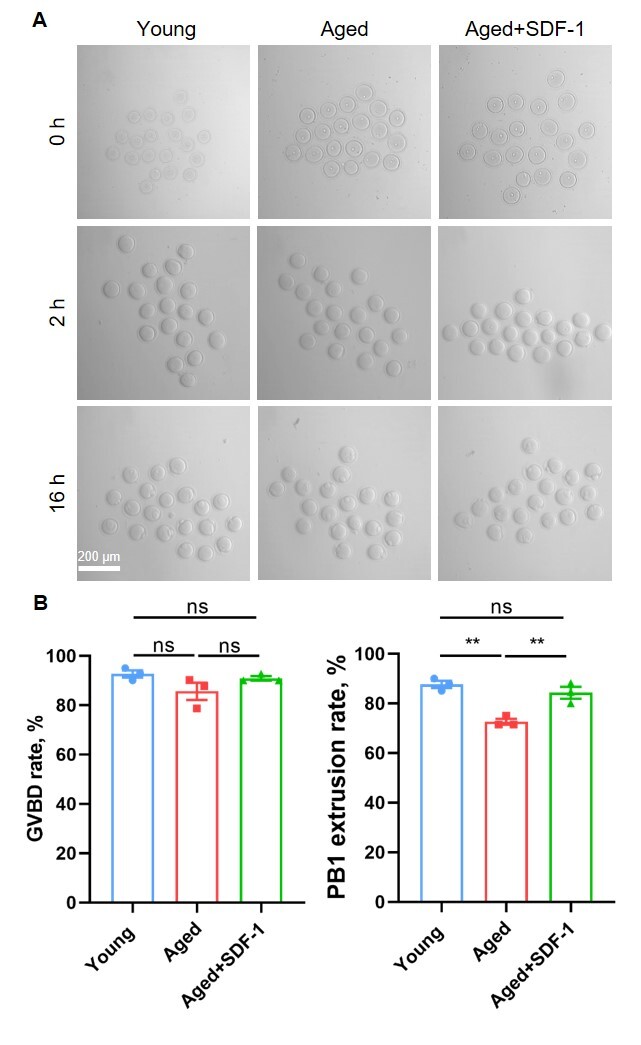

Supplement: Supplementary file 2 — Supporting File 2: advs76902‐sup‐0002‐FigureS1‐S4.zip. [file ADVS-9999-e76902-s001.zip › fig s1_300dpi.jpg]

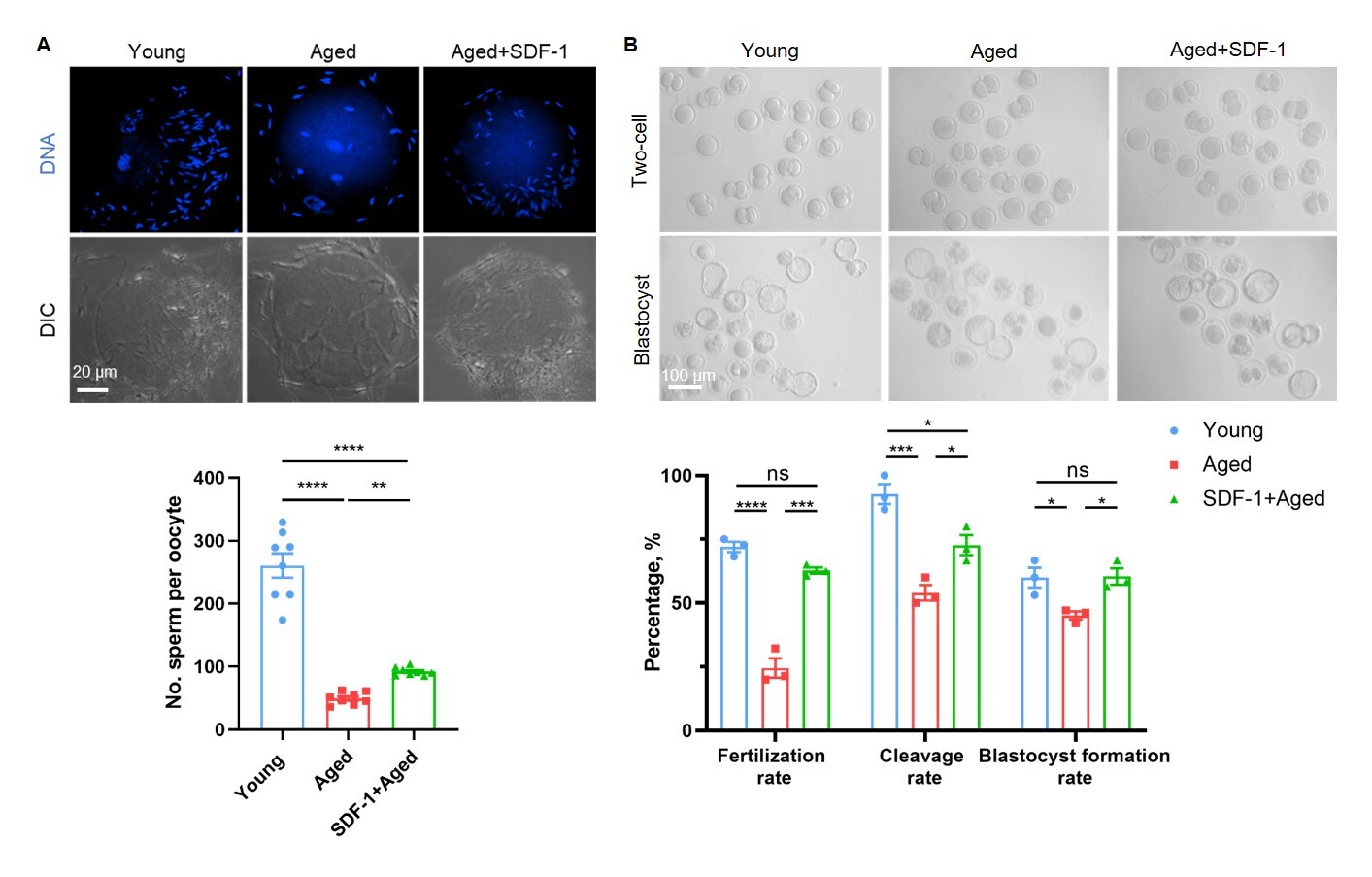

Supplement: Supplementary file 2 — Supporting File 2: advs76902‐sup‐0002‐FigureS1‐S4.zip. [file ADVS-9999-e76902-s001.zip › fig s2_300dpi.jpg]

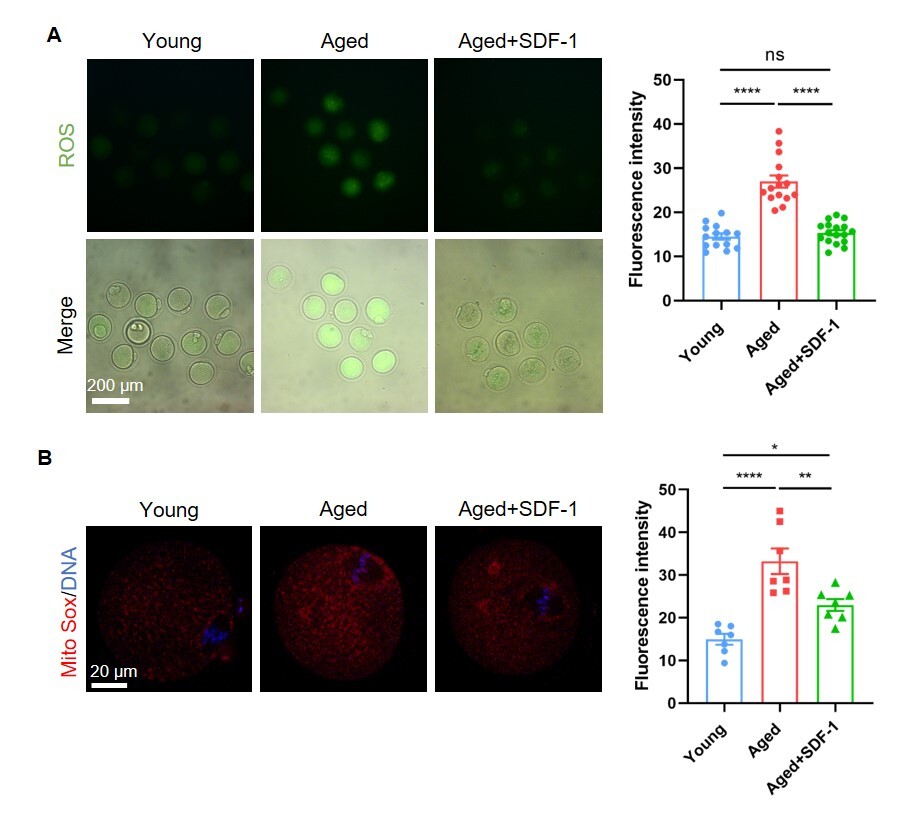

Supplement: Supplementary file 2 — Supporting File 2: advs76902‐sup‐0002‐FigureS1‐S4.zip. [file ADVS-9999-e76902-s001.zip › fig s3_300dpi.jpg]

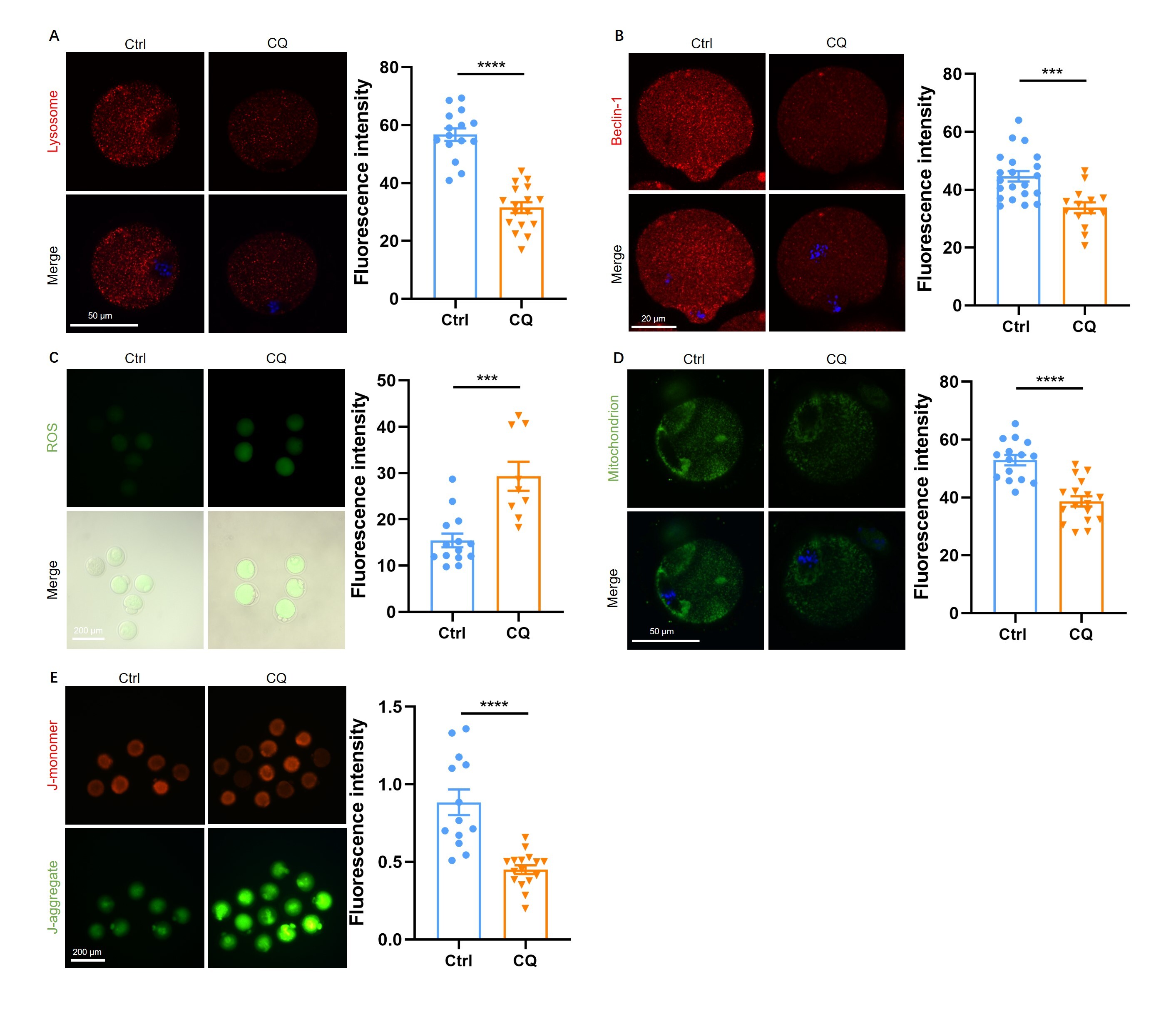

Supplement: Supplementary file 2 — Supporting File 2: advs76902‐sup‐0002‐FigureS1‐S4.zip. [file ADVS-9999-e76902-s001.zip › fig s4_300dpi.jpg]
